# Relationship between ball release point variability and pitching performance in major league baseball

**DOI:** 10.3389/fspor.2024.1447665

**Published:** 2024-11-18

**Authors:** Kazuki Wakamiya, Hideaki Nagamoto, Ryusei Yamaguchi, Takumi Okunuki, Toshihiro Maemichi, Zijian Liu, Yuki Ogawa, Yusuke Kobayashi, Tsukasa Kumai

**Affiliations:** ^1^ Graduate School of Sport Sciences, Waseda University, Saitama, Japan; ^2^ Institute for Sport Sciences, Waseda University, Saitama, Japan; ^3^ Department of Orthopaedic Surgery, School of Medicine, Tohoku University, Miyagi, Japan; ^4^ Research Organization of Science and Technology, Ritsumeikan University/Research Fellow of Japan Society for the Promotion of Science, Tokyo, Japan; ^5^ Faculty of Sport Sciences, Waseda University, Saitama, Japan

**Keywords:** baseball, movement variability, pitching, release point, tracking data

## Abstract

**Introduction:**

This study examined the relationship between ball release points and pitching performance among professional baseball pitchers, with a focus on variability.

**Methods:**

We used open-source data to compare ball release point variability between Major League Baseball (MLB) and Minor League Baseball (MiLB) players. The relationship between pitching performance and variability was analyzed using multiple regression analysis.

**Results:**

MLB players exhibited smaller ball release point variability compared to MiLB players. The analysis showed that pitching performance was strongly related to ball release point variability, especially in the horizontal direction on the coronal plane. Horizontal ball release point variability was most strongly related to strikeout ability among pitching performances.

**Discussion:**

These results suggest that reducing horizontal ball release point variability may improve pitching performance, particularly by increasing strikeouts and reducing home runs allowed. This study provides a data-driven approach to understanding the mechanics of pitching and can be applied to the development of advanced training methods and technical solutions aimed at improving pitching performance in baseball players.

## Introduction

Recent technological developments have made it easier to obtain tracking data such as ball spin (rotations per minute: RPM) and ball release point. In Major League baseball (MLB) and Minor League Baseball (MiLB), these data are used to evaluate player performance and to improve performance through coaching. While tracking data are widely used at the field level, academic research is still limited. MLB tracking data are available online and have been used in several studies. Many studies using tracking data have investigated the association with pitching injuries such as ulnar collateral ligament (UCL) injury (1–4). Changes in horizontal ball release point are considered a risk factor for UCL injury, and several studies have focused on the ball release point (3, 4).

On the other hand, few studies have reported an association with ball release point and pitching performance. Studies in MLB pitchers have reported that pitch velocity, ball release point variability, range of velocities on all pitches, and horizontal ball release point are predictors of pitching performance (5). This study evaluated two types of ball release point variability: ball release point variability of multiple pitch types combined and ball release point variability of individual pitch types. However, the importance of throwing multiple pitch types with the same ball release point is unclear because the ball release point variability of multiple pitch types is affected by the variability of each pitch type. Therefore, we consider it necessary to examine the rate of match between 4-seam and the ball release point on the breaking ball. Moreover, only ball release points on the coronal plane have been considered in the study focusing on ball release points. This is because ball release point data on the sagittal plane was newly published in 2016, and the number of data available is smaller than for other variables (6). In light of the fact that ball release point variability on the coronal plane is a factor in pitching performance, it is possible that variability on the sagittal plane is a factor in performance as well.

It has also been reported that the variability of pitching form decreases as the level of competition increases (7). Based on these facts, it is considered important to throw with the same pitching form. However, in a study that examined pitching form variability between different levels of competition, only five pitches were used, which is a small number of trials to examine differences in variability (8). Hence, we believe that the use of tracking data sites that can obtain large amount of pitching data will allow us to fully examine the variability in ball release point variability between different levels of competition.

Historically, Earned Run Average (ERA) has been used to evaluate pitcher performance. However, ERA is influenced by the defensive abilities of fielders, leading to potential inaccuracies in assessing a pitcher's true skill. To address this issue, Fielding Independent Pitching (FIP) has been increasingly adopted. FIP is calculated using only strikeouts, walks, and home runs, excluding the impact of fielding abilities (9). This metric is widely used in several studies to evaluate performance (10, 11). However, FIP also has limitations, as it can be affected by the size of the ballpark, influencing home run outcomes. Therefore, an adjusted metric, Expected Fielding Independent Pitching (xFIP), was introduced. xFIP accounts for these variations by normalizing home runs (12). Based on this background, xFIP is used as the pitching performance metric for this study. Improving xFIP means improving strikeouts, walks, home runs allowed, or all of the above. Since different pitchers have different problems, such as low strikeouts, high walks allowed, and high home runs allowed, it is important to examine each problem individually. Therefore, in this study, K/9 (Number of strikeouts per 9 innings), BB/9 (Number of walks per 9 innings), and HR/9 (Number of home runs per 9 innings) are used as indices to evaluate the individual components of xFIP. By evaluating these measures, the relationship between release point variability and pitching performance can be clarified in more detail.

Therefore, the relationship between ball release point variability and pitching performance should be examined, including ball release point data on the sagittal plane. To achieve this, we examined the differences in ball release point variability between MLB and MiLB players, as well as the relationship between ball release point variability and pitching performance metrics (xFIP, K/9, BB/9, and HR/9) in MLB players.

The hypotheses are as follows:

1. The ball release point variability of MLB players is smaller than that of MiLB players.
2. Smaller ball release point variability is associated with higher xFIP values, and in particular, better BB/9 performance.

## Materials and methods

### Sample

The subjects were 344 MLB players and 64 MiLB players who pitched as starters from 2021 to 2023. The number of pitches analyzed were as follows: for MLB pitchers, 300,884 four-seam and 517,530 breaking balls; for MiLB pitchers, 42,585 four-seam and 77,440 breaking balls. The selection criteria were that the players must have pitched at least 1,500 pitches in a season, that at least 70% of the games they pitched were as starters, and that the percentage of 4-seam pitches was greater than 5%.

### Procedure

Basic information and performance data for each pitcher were obtained from FanGraphs (13), and tracking data were obtained from Baseball Savant (14). Basic information and performance data for each pitcher were obtained from the former, and tracking data were obtained from the latter. The basic information obtained was height, weight, and age.

The performance data obtained were xFIP, K/9 (Number of strikeouts per 9 innings), BB/9 (Number of walks per 9 innings), and HR/9 (Number of home runs per 9 innings). Due to the difficulty in performance evaluation across leagues with varying competitive levels from A to AAA in MiLB, the performance evaluation was limited to MLB players only. Additionally, the analysis was conducted under the assumption that MLB players have higher pitching performance than MiLB players, given that MLB is a higher competitive level league than MiLB.

**Figure 1:**
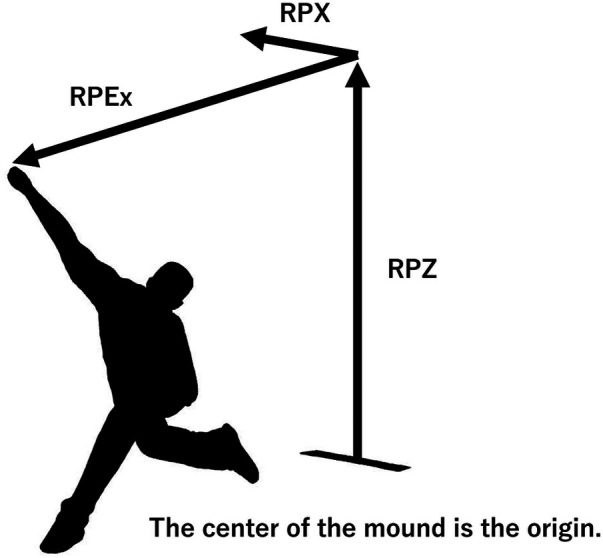
Image of the ball release point and its origin point.

**Figure 2:**
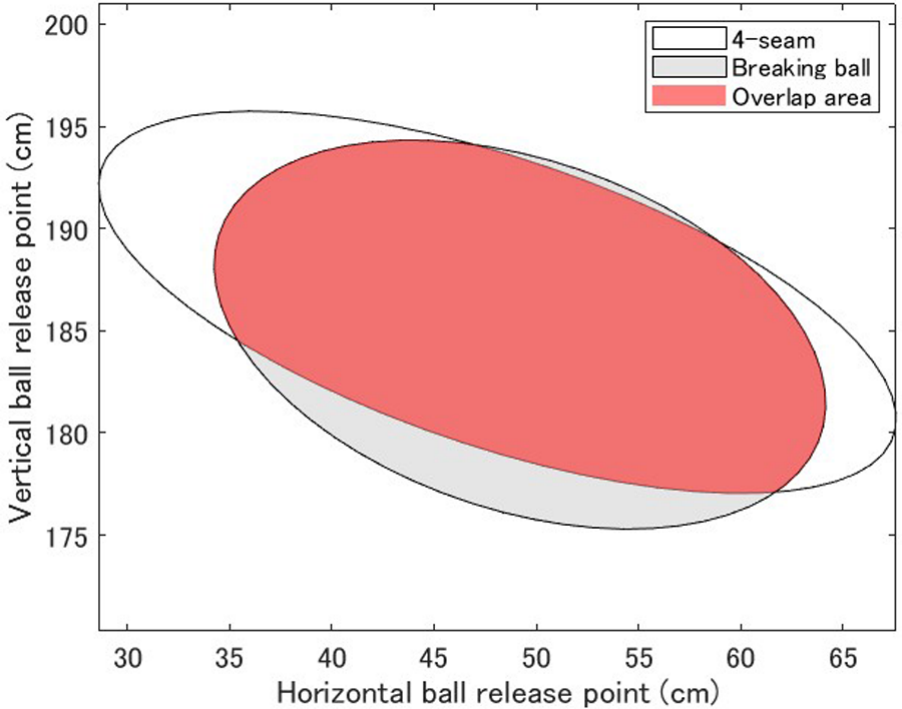
Overlap of 95% confidence ellipse.

Tracking data obtained were pitch speed, pitch type, horizontal release point on the coronal plane (RPX), vertical release point (RPZ), and release extension (RPEx), where RPX is the width from the mound to the ball release point in the coronal plane. RPEx is the length from the mound to the release point in the sagittal plane (Figure 1). The measurement error of the release point is reported to be less than 1.02 cm (2). The ball release points were divided into two groups: data for 4-seam only, and data for other breaking ball data except for 4-seam. Ball release point variability was calculated using 95% confidence ellipses (5). The 95% confidence ellipses were ellipses on the coronal plane consisting of RPX and RPZ, and ellipses on the sagittal plane consisting of RPEx and RPZ, for a total of four ellipses for the 4-seam and breaking balls (Figure 2). The width, height, and area of each ellipse were calculated and used as a measure of variability. The width of the ellipse on the coronal plane is the RPX variability, the width of the ellipse on the sagittal plane is the RPEx variability, and the height of the ellipse is the RPZ variability. The center point of the ellipse is the mean value of each release point. In addition, the percentage of ball release point match between the 4-seam and the breaking ball was calculated as Overlap%. The equation was as follows.

$$\mathrm{Overlap}\text{\%}\;=\;\frac{\mathrm{area}\;\mathrm{where}\;\mathrm{the}\;\mathrm{two}\;\mathrm{ellipses}\;\;\mathrm{overlap}}{\mathrm{area}\;\mathrm{where}\;\mathrm{the}\;\mathrm{two}\;\mathrm{ellipses}\;\mathrm{overlap}+\;\mathrm{area}\;\mathrm{where}\;\mathrm{the}\;\mathrm{two}\;\mathrm{ellipses}\;\mathrm{do}\;\mathrm{not}\;\mathrm{overlap}}$$

### Statistical analysis

For the comparison of MLB and MiLB players, each tracking data was compared using a *t*-test or Mann-Whitney *U*-test. Multiple regression analysis was used to examine the relationship between pitching performance and tracking data. The dependent variable was the xFIP and the independent variable was each tracking data. In addition, if the predictive model was determined to be significant, a further multiple regression analysis was performed with the predictive model variable as the independent variable and K/9, BB/9, and HR/9 as the dependent variables, respectively.

## Results

Height, weight, and age of the MLB players were significantly higher, heavier, and older than MiLB players (Table 1).

**Table 1:** Mean of height, body weight, and age in Major league baseball (MLB) and Minor league baseball (miLB).

	Mean (SD)		*p*-value
	MLB	MiLB	
Height (cm)	190.74 (5.30)	188.28 (4.93)	<0.001
Body weight (kg)	98.18 (11.99)	94.21 (10.53)	0.003
Age	28.58 (4.10)	26.36 (3.76)	<0.001

For the tracking data, the MLB players had significantly faster pitch velocities. In the coronal plane, the variability of RPX, RPZ, and ellipse area were significantly smaller in MLB players for both 4-seam and breaking ball. In the sagittal plane, the variability of RPZ was significantly smaller in MLB players for both 4-seam and breaking ball. Four-seam and breaking ellipse match rates were not significantly different between MLB and MiLB players (Table 2).

**Table 2:** Mean of velocity, ball release point and ball release point variability.

	Major league baseball (MLB)	Minor league baseball (MiLB)	*p*-value
	Mean ± SD	Mean ± SD	
Velocity (km/h)	150.32 ± 3.53	147.28 ± 3.32	<0.001
Four-seam			
RPX mean (cm)	55.22 ± 18.22	55.46 ± 20.18	0.924
RPZ mean (cm)	181.19 ± 12.36	178.10 ± 10.88	0.062
RPEx mean (cm)	195.31 ± 12.32	190.04 ± 12.38	0.002
RPX variability (cm)	30.60 ± 12.29	35.21 ± 16.17	0.014
RPZ variability in coronal plane (cm)	15.21 ± 2.52	17.48 ± 3.43	<0.001
RPZ variability in sagittal plane (cm)	15.70 ± 3.06	17.30 ± 3.22	<0.001
RPEx variability (cm)	25.41 ± 4.57	24.58 ± 4.56	0.195
Ellipse area in coronal plane (cm^2^)	373.50 ± 184.81	497.06 ± 300.81	<0.001
Ellipse area in sagittal plane (cm^2^)	317.42 ± 101.58	340.62 ± 112.92	0.111
Breaking ball			
RPX mean (cm)	57.51 ± 18.01	58.53 ± 20.14	0.727
RPZ mean (cm)	179.75 ± 12.31	175.98 ± 11.46	0.021
RPEx mean (cm)	193.99 ± 11.91	189.12 ± 12.07	0.002
RPX variability (cm)	35.39 ± 15.24	39.54 ± 16.77	0.002
RPZ variability in coronal plane (cm)	16.83 ± 3.13	19.47 ± 3.89	<0.001
RPZ variability in sagittal plane (cm)	18.10 ± 4.11	19.99 ± 3.94	<0.001
RPEx variability (cm)	29.93 ± 8.39	29.24 ± 7.08	0.610
Ellipse area in coronal plane (cm^2^)	471.00 ± 287.70	625.54 ± 352.58	<0.001
Ellipse area in sagittal plane (cm^2^)	441.03 ± 250.76	468.65 ± 179.64	0.025
Overlap			
Overlap% in coronal plane (%)	58.68 ± 19.48	62.18 ± 19.01	0.098
Overlap% in sagittal plane (%)	59.69 ± 16.26	61.90 ± 16.79	0.285

RPX stands for horizontal ball release point variability. RPY stands for vertical ball release point variability. RPEx stands for antero-posterior ball release point variability.

The following predictors of xFIP were identified in MLB players: pitch speed, variability of RPX in 4-seam, mean RPEx in 4-seam, variability of RPEx in 4-seam, and mean RPZ in 4-seam. The coefficient of determination for the predictive model was 0.207 (Table 3). In terms of the association between the five extracted variables and K/9, BB/9, and HR/9, K/9 was the most strongly associated with the five variables. Pitch velocity was extracted as a predictor of BB/9, but with a coefficient of determination of only 1.4%. Variability of RPX in 4-seam, pitch velocity, and variability of RPEx in 4-seam were identified as predictors of HR/9 (Table 4).

**Table 3:** Multiple regression analysis for pitching performance.

Independent variable	β	*r*	*p*-value
Velocity	−0.398	−0.413	<0.001
RPX variability of 4-seam	0.161	0.174	0.002
RPEx mean of 4-seam	−0.097	−0.144	0.051
RPEx variability of 4-seam	0.081	0.060	0.103
RPZ mean of 4-seam	0.070	0.099	0.155

Pitching performance was evaluated using xFIP (an index consisting of strikeouts, earned runs allowed, and home runs allowed). RPX stands for horizontal ball release point variability. RPY stands for vertical ball release point variability. RPEx stands for antero-posterior ball release point variability.

R^2^ = 0.207. β = standardized beta coefficient. *r* = Pearson product-moment correlation.

**Table 4:** Multiple regression analysis for K/9, BB/9 and HR/9.

Independent variable	β	*r*	*p*-value
K/9			
Velocity	0.514	0.544	<0.001
RPZ mean of 4-seam	−0.137	−0.191	0.002
RPEx mean of 4-seam	0.141	0.229	0.002
RPX variability of 4-seam	−0.122	−0.122	0.006
BB/9			
Velocity	0.118	0.118	0.029
HR/9			
RPX variability of 4-seam	0.168	0.190	0.002
Velocity	−0.174	−0.168	<0.001
RPEx variability of 4-seam	0.128	0.146	0.016

K/9 is number of strikeouts per 9 innings. BB/9 is number of walks per 9 innings. HR/9 is number of home runs per 9 innings. RPX is horizontal ball release point variability. RPY is vertical ball release point variability. RPEx is antero-posterior ball release point variability.

K/9: R^2^ = 0.345. BB/9: R^2^ = 0.011. HR/9: R^2^ = 0.072.

β = standardized beta coefficient.

*r* = Pearson product-moment correlation.

## Discussion

The purpose of this study was to compare ball release point variability between MLB and MiLB pitchers and to determine the relationship between ball release point variability and pitching performance. In the comparison of MLB and MiLB players, MLB players had significantly faster pitch velocities. Furthermore, the MLB players had significantly smaller RPX variability, RPZ variability, and ellipse area on the coronal plane for both the 4-seam and breaking ball pitches. The results of the multiple regression analysis indicated that several factors, including pitch velocity, RPX variability, RPEx variability, RPZ mean value, and RPEx mean value, serve as significant predictors of xFIP. Notably, within the components of xFIP, pitch velocity, RPX variability, RPZ mean value, and RPEx mean value were significant predictors for K/9, while pitch velocity, RPX variability, and RPEx variability were significant predictors for HR/9.

### Pitch velocity

MLB players' pitch velocities from this study were similar to those from 2020 s studies (15), but faster than those from 2010 s studies (5, 16–18), This can be attributed to the fact that MLB's average pitch speed has increased over the years (19). The results of the present study confirm that MLB players have faster pitch speeds than MiLB players, and that pitch speed increases as the level of competition increases.

Ball speed was also identified as a predictor of xFIP in MLB, explaining 16.9% of the variance in xFIP. If the other variables remain constant, a 1 km/h increase in pitch speed results in a 0.398 decrease in xFIP. Although FIP is used as the pitching performance measure in the previous study, the variance explained by pitch speed was 10.4% (5). Based on these results, it is possible that the importance of pitch speed has become more significant due to the recent increase in speed in MLB. Furthermore, pitch speed was most strongly associated with K/9 among K/9, BB/9, and HR/9. Pitch speed explained 29.4% of the variance in K/9, indicating that faster pitch speeds may improve strikeout ability, leading to better xFIP. Additionally, while pitch speed was not as strongly correlated with HR/9 as with K/9, it still demonstrated a notable association. Higher pitch speeds could lead to fewer home runs, as batters may miss high-velocity pitches even when the ball is in a typically home-run-prone zone. This reduction in home runs also contributes to a better xFIP. On the other hand, pitch speed negatively impacted BB/9, although it only explained 1% of the variance, indicating a very minor influence. Overall, increasing pitch speed is likely to enhance pitching performance by improving strikeout ability and reducing home runs.

### Variability of ball release point

Ball release point variability was significantly lower for both the 4-seam and the breaking ball, as were RPX and RPZ variability and ellipse area on the coronal plane for MLB players. These results support previous research showing less variability in pitching form at higher levels of competition (7). In particular, the elliptical area of the 4-seam on the coronal plane was about 100 cm^2^ larger for MLB players compared to MiLB players, suggesting that it may be important to improve ball reproducibility by throwing one pitch type with the same ball release point. The difference in variability of RPZ between MLB and MiLB players was about 2.5 cm for both 4-seam and breaking ball pitches. In contrast, the difference in variability of RPX was about 5 cm for both the 4-seam and breaking ball pitches, about twice as large as the difference in variability of RPZ. This is similar to previous studies (9), which suggest that ball release points are more likely to vary horizontally.

RPX variability was also identified as a predictor of xFIP, whereas RPZ variability was not. Variability in RPX is the second-best predictor of xFIP next to pitch speed, with xFIP improving by 0.161 for every 1 cm decrease in RPX variability. Furthermore, variability in RPX was associated with K/9 and HR/9. Since horizontal ball release point on coronal plane has been reported to be a predictor of pitch location (20), it is possible that greater horizontal variability on coronal plane may lead to more missed pitches and more home runs allowed, and fewer strikeouts. Therefore, it is thought that a decrease in RPX variability can improve xFIP by increasing strikeouts and reducing home runs allowed. In addition, variability in RPEx was also found to be a weak predictor of HR/9. Ball release point consistency was related to the covariation of each joint movement. In particular, the covariation between ankle and knee joint angles has been reported to play a large role in the stride leg (21). The variability of RPEx may also be related to the covariation of the knee and ankle joints, since the variability of the knee and hip joint positions is reduced when the covariation works. It is possible that when the covariation does not work well, the variability of RPEx also increases, leading to more missed pitches and more homeruns allowed. Therefore, it is considered that a decrease in the variability of RPEx leads to a decrease in the home runs allowed and an improvement in xFIP. However, the variability of RPEx has not differed between MLB and MiLB players, and it does not explain as much variance in xFIP as in variability of RPX. Thus, within players at higher competitive levels, variability in RPEx may make it difficult to see differences. On the other hand, there was no significant difference in the elliptical match rate between the 4-seam and the breaking ball. It has been reported that different pitch types have different release points (22), suggesting that it may not be important to throw the 4-seam and the breaking ball at the same ball release point. Hence, considering xFIP and the metrics that make it up, the smaller the variability in RPX and RPEx, the better the pitching performance.

### Average of ball release point

The smaller 4-seam average of RPZ and larger RPEx average were also predictors of K/9. The concept of Vertical Approach Angle (VAA) has gained attention in recent years. VAA refers to the vertical angle at which the ball approaches home base. The closer the ball's trajectory is to being level with the ground, the higher the likelihood of a strikeout (23). Considering VAA, K/9 is thought to improve as the RPZ decreases and the RPEx increases, bringing the VAA closer to horizontal. In addition, RPZ and RPEx may be influenced by height, with MLB players having a higher release point compared to MiLB players. While this difference might be attributed to height, since a lower release point correlates with better strikeout ability and pitching performance, these results suggest that height difference is not a major factor. Lowering the RPZ can be achieved by adjusting the arm angle and swinging the arm horizontally. However, if lowering the arm angle were effective, a higher average RPX should correlate with a higher K/9, which our data did not support. Therefore, lowering the arm angle does not improve strikeout ability. Another method would be to increase the hip flexion angle or trunk forward tilt angle at ball release. Since hip range of motion has been reported to be associated with pitching injuries (24), hip flexibility may be important for both high strikeout ability and prevention of pitching injuries. Therefore, the average RPZ and RPEx values were significantly related to strikeout ability, making them substantial predictors of xFIP.

The results of this study clearly show that ball release point is related to pitching performance, especially to strikeout ability. These results contribute to the improvement of pitching performance in pitcher development. Further research may provide more insight into pitcher development using tracking data.

### Limitations

There are several limitations to this study. One is that the study grouped all breaking ball pitches into a single category and was unable to examine differences among pitch types. Another is that it was unable to consider variables such as the number of spins, axis of spin, and amount of change in the ball. Because MLB pitchers throw different types of pitches, and because the percentage of pitches thrown by each type of pitcher varies widely, we declined to examine each type of pitch in this study. In addition, the axis of spin and the amount of change in the ball were excluded from the analysis in this study because they may be affected by the release point. Since release parameters and ball velocity alone explained only 20% of the variance in xFIP, further study is needed to add these items. Moreover, since biomechanical data could not be obtained, the relationship between tracking data and biomechanical data is unclear. It is important to clarify the relationship between tracking data and biomechanical data in the future. Additionally, since MiLB includes leagues with varying competitive levels from A to AAA, it was not possible to directly examine the relationship between pitching performance and these different levels within MiLB. However, it is crucial to focus on achieving success in MLB, and MiLB players should aim to improve their pitching performance to succeed at the MLB level.

## Conclusion

The results of this study revealed a relationship between ball release point variability and pitching performance. Reducing variability in horizontal release points in the coronal plane and antero-posterior release points in the sagittal plane can improve strikeout ability and reduce home run allowed, thus enhancing overall pitching performance. It was also shown that the 4-seam release point was lower and closer to the batter, potentially improving strikeout ability and enhancing overall pitching performance.

## Data Availability

The original contributions presented in the study are included in the article/Supplementary Material, further inquiries can be directed to the corresponding author.
